# Prevalence and Determinants of Depression, Anxiety, and Stress Among Secondary School Students

**DOI:** 10.7759/cureus.44182

**Published:** 2023-08-26

**Authors:** Mohammed M Barnawi, Ali M Sonbaa, Maysa M Barnawi, Abdullah H Alqahtani, Bashaier A Fairaq

**Affiliations:** 1 Postgraduate Training Program for Preventive Medicine, Ministry of Health, Taif, SAU; 2 Psychiatry, Imam Abdulrahman Bin Faisal University/King Fahd Hospital of the University, Al Khobar, SAU; 3 Community Medicine, College of Medicine, Umm Al-Qura University, Makkah, SAU; 4 Psychiatry, Medicine Department, Johns Hopkins Aramco Healthcare (JHAH), Dhahran, SAU; 5 Population Health Management Administration, Makkah Health Cluster, Makkah, SAU

**Keywords:** social stressors, saudi arabia, mental health, dass-21, bullying, adolescents

## Abstract

Background: Adolescence represents a critical period characterized by extensive changes across various domains. While some of the changes and behaviors that emerge during this period can have detrimental effects on adolescents' present and future health, mental health disorders continue to constitute a leading cause of morbidity among young individuals.

Objective: Assess the prevalence and determinants of depression, anxiety, and stress among secondary school students in Saudi Arabia.

Methods: This analytical cross-sectional study utilized an online questionnaire to collect responses from secondary school students in Saudi Arabia. The Arabic version of the Depression, Anxiety, and Stress Scale - 21 Items (DASS-21) was used to measure depression, anxiety, and stress levels. Data were analyzed using IBM Statistical Package for the Social Sciences software (SPSS, version 29.0, IBM Corp., Armonk, NY, USA). The study included 702 participants.

Results: The prevalence of anxiety was 35.2%, followed by depression (30.8%) and stress (14.7%). Females had significantly higher anxiety and stress scores than males (P=0.004, and P=0.011, respectively). Students who were bullied in the last 30 days had significantly higher depression, anxiety, and stress scores than those who were not bullied (P<0.001 for all). Participants who experienced physical assault in the last 12 months had significantly higher depression, anxiety, and stress scores than those who did not (P<0.001 for all). Participants who had had fights in the last 12 months had significantly higher depression (P=0.004) and anxiety (P<0.001) scores than those who did not. Participants who felt unsafe on the way to school had significantly higher depression, anxiety, and stress scores than those who did not (P<0.001 for all).

Conclusion: The study revealed prevalence rates of depression (30.8%), anxiety (35.2%), and stress (14.7%) among secondary school students in Saudi Arabia. The study highlights the importance of addressing social stressors such as bullying and physical assault and promoting a safe and supportive school environment to prevent mental health disorders in this population.

## Introduction

Adolescence is a period of remarkable change on all levels, biological, physiological, and psychological [1]. Unfortunately, some of the changes and behaviors that appear during this period can be detrimental to adolescents' current and future health [2]. The importance of this age group is emphasized in the fact that adolescents represent a high proportion of the total population. In fact, those under 25 years old constitute 39.4% of the population in the Saudi Arabia region [3]. In fact, in Saudi Arabia, one-fifth of the population is between 10-19 years [3].

Globally, mental health disorders are a leading cause of morbidity in young people [4]. Such disorders put young people at risk of smoking, substance abuse, violence, lower academic achievement, and suicide. Although these disorders may develop during adolescence, they usually persist well into adulthood, negatively impacting the lives of those affected by them [5,6]. Evidence suggests that preventive interventions can be applied to young people to reduce the cost attributable to these disorders [7]. Depression and anxiety are two of the primary culpable disorders [8]. Depression is a mood disorder characterized by sadness, emptiness, or irritability, and associated with physical and cognitive changes that hinder the ability to function [9]. The distinguishing feature of all anxiety disorders is "excessive fear and anxiety followed by avoidance of an object or situation that poses no danger" [10]. Stress on the other hand is represented in feelings of emotional strain and unease when faced with challenging situations [11].

According to "Maternal, Child and Adolescent Mental Health," a technical paper from the World Health Organization (WHO), the estimated prevalence of mental health disorders in adolescents is 15%-36% [12]. An analysis of data from the Global School-based Student Health Survey (GSHS) in 19 low- and middle-income countries, including the United Arab Emirates, Jordan, Lebanon, and Morocco, found that 35% of all participating students had symptoms of depression [13]. In Saudi Arabia, the estimated prevalence of depression was reported to be 30% in Riyadh city [14]. Another study in Qassim found that among students, 34% were mildly depressed, 24.6% were moderately depressed, 10.4% were severely depressed, and 5% were extremely severely depressed. In regard to anxiety, 34.1% had mild anxiety, 19.5% had moderate anxiety, and 9.8% had severe anxiety [15].

Mental health disorders are on the rise, and the consequences they can have on young people are monumental. Literature shows that depression and anxiety are the most common disorders in this age group. However, data in the Arab world are still lacking. In Saudi Arabia, different studies show a wide range of estimates for the prevalence of depression and anxiety. Furthermore, the significance of some of the determinants showed variability between studies, and others were not explored in different populations. Therefore, we conducted the present study to add to the body of evidence in order to inform public policies and to develop and implement culturally appropriate preventive interventions. The current study aimed to assess the prevalence and determinants of depression, anxiety, and stress among secondary school students in Saudi Arabia.

## Materials and methods

Study design

This study employed an analytical cross-sectional design, utilizing an online questionnaire to collect responses from secondary school students in Saudi Arabia. The Arabic version of the Depression, Anxiety, and Stress Scale - 21 Items (DASS-21) [16], a validated tool for measuring depression, anxiety, and stress levels, was utilized.

Study population

The study included male and female secondary school students in Saudi Arabia. The inclusion criteria comprised all secondary school students in Saudi Arabia, encompassing students from both governmental and private schools. The exclusion criteria encompassed students attending evening schools, special-needs education schools, and students with intellectual disabilities.

Using Epi Info 7 software (developed by the Centers for Disease Control and Prevention (CDC), Atlanta, Georgia, USA), a minimum sample size of 384 secondary school students was determined. Assuming a prevalence of 50% for depression, anxiety, and stress, with a precision of 5% and a confidence level of 95%, the final sample size was set at approximately 450 students, accounting for a 20% non-response rate. Convenient sampling was employed to recruit the participants. In the present study, we have collected a substantial total of 702 responses to enhance the study's statistical power and achieve a more comprehensive representation at a national level.

Data collection

Data were collected using a self-administered questionnaire that utilized validated tools to measure rates of depression, anxiety, stress, and health behaviors among students. In addition to the adopted tools, age, gender, self-reported height, and weight were obtained from the participants. The target population was reached using a social media platform (WhatsApp) using a link to the online questionnaire. The data collection took place during June 2023.

The Global School-Based Student Health Survey (GSHS), a self-administered questionnaire developed by the WHO, was used to obtain data on young people's health behavior and protective factors related to morbidity and mortality [16]. We used a modified version of the GSHS questionnaire based on a review of the literature and expert opinions. Behavioral questions included sedentary leisure behavior, frequency of hunger, sleep during the weekdays, frequency and type of bullying, frequency of physical attacks and fights, and absenteeism due to safety concerns.

The Depression, Anxiety, and Stress Scale-21 (DASS-21) was used to measure negative emotional states of depression, anxiety, and stress [17] (Appendix A). The anxiety scale corresponded to symptom criteria for anxiety disorders, except for generalized anxiety disorder. The depression scale corresponded to mood disorders, and the stress scale corresponded to the Diagnostic and Statistical Manual of Mental Disorders, Fifth Edition (DSM-IV) symptom criteria for general anxiety disorder (GAD). Subjects were asked to rate the extent to which they had experienced a group of states associated with the three disorders over the past week using four-point severity and frequency scales.

Statistical analysis

The data was analyzed using the Statistical Package for the Social Sciences software (IBM Corp., released in 2021; IBM SPSS Statistics for Macintosh, Version 29.0; Armonk, NY: IBM Corp.). Continuous variables were assessed for normal distribution using the Shapiro-Wilk test. Since the test revealed that all continuous variables were skewed, median and interquartile range (IQR) were used. Categorical variables were presented in frequency tables and percentages.

The outcome variables were the total score of the DASS-21 components, which included depression, anxiety, and stress (Appendix B). The total scores were calculated by summing the subscale items. The components were further categorized as normal, mild, moderate, severe, and extremely severe for descriptive statistics. For continuous variables, the strength of the association was determined using Spearman's correlation coefficient. Differences in DASS-21 subscales between groups of categorical variables were tested using non-parametric tests, including the Mann-Whitney and Kruskal-Wallis tests, as appropriate.

Linear regression analysis was conducted following the assessment of three outcome variables: depression, anxiety, and stress total scores. Normal distribution was presumed, with skewness ranging from (-1) to (1). Variables displaying significant associations with the total scores were subjected to both forward and backward linear regression while ensuring validation of the multicollinearity assumption. Among the various models tested, the most optimal models for each outcome were identified and reported. Statistical significance was established at a threshold of a P-value < 0.05.

Ethical considerations

The study protocol was approved by the research ethics committee of the Research and Studies Department of the Directorate of Health Affairs, Taif, Saudi Arabia (approval number 550), and all participants were informed about the study's purpose. Written consent was obtained, and confidentiality was assured. Participation was voluntary, and participants were free to withdraw from the study at any time. There was no conflict of interest. All responses were anonymous and used solely for research purposes. The current study adhered to the Strengthening the Reporting of Observational Studies in Epidemiology (STROBE) guidelines to ensure comprehensive and transparent reporting of the observational research.

## Results

The median age of participants was 18 years, with an IQR of 17-18. The median body mass index (BMI) was 22.27 kg/m^2^, with an IQR of 19-26.14. Among the participants, 79.5% were females and 20.5% were males. The majority of participants were in their third year of school (70.7%). The majority of participants had a normal BMI (46.5%). The detailed results of sociodemographic characteristics are captured in Table 1.

**Table 1:** Sociodemographic characteristics of secondary school students in Saudi Arabia

Variable	Categories	Number	Percentage
Gender	Female	558	79.5%
	Male	144	20.5%
School year	First	79	11.3%
	Second	127	18.1%
	Third	496	70.7%
Body mass index (BMI)	Underweight	144	21.2%
	Normal	316	46.5%
	Overweight	147	21.6%
	Obese	72	10.6%

**Figure 1:**
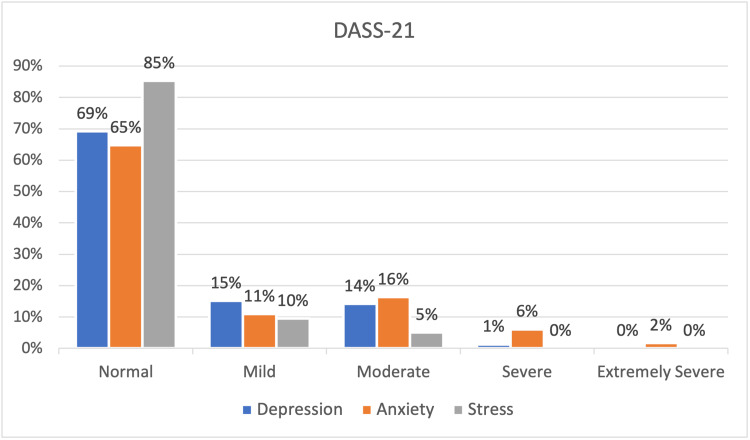
Levels of depression, anxiety, and stress among secondary school students according to DASS-21 in Saudi Arabia DASS-21: Depression, Anxiety, and Stress Scale - 21 Items (DASS-21)

Excluding students who had normal scores in the subscales of DASS-21, anxiety had the highest prevalence at 35.2%, followed by depression at 30.8%, and stress at 14.7%. Notably, 7.8% of students showed severe or extremely severe levels of anxiety, while 1.3% showed severe or extremely severe levels of depression. None of the students showed severe or extremely severe levels of stress. The levels of depression, anxiety, and stress are demonstrated in detail in Figure 1.

Of the participants, 32.1% reported never feeling hungry due to a lack of food at home. The majority of participants (43.4%) spent seven to eight hours sitting during a typical day, while only 7.1% spent less than an hour. Regarding sleep duration, 16.7% of participants slept for five or six hours, while 14.8% slept for seven or eight hours. The majority of participants (77.6%) reported not being bullied in the last 30 days. Of the total sample, most reported being bullied for their body or face (8%). More than one-third (35.5%) of participants reported receiving classes on how to prevent being bullied during the current study year. The results of the social stressors are shown in Table 2.

**Table 2:** Frequency of social and peer stressors among secondary school students in Saudi Arabia

Question	Answers	Number	Percentage
During the last 30 days, how many times did you feel hungry because there wasn't enough food at home?	Never	225	32.10%
	Rarely	173	24.60%
	Sometimes	163	23.20%
	Most of the time	79	11.30%
	Always	62	8.80%
During the usual day, how long do you spend watching TV, using the computer, chatting with friends, or doing something that requires you to be sitting?	<1 hour	50	7.10%
	1-2 hours	64	9.10%
	3-4 hours	102	14.50%
	5-6 hours	181	25.80%
	7-8 hours	305	43.40%
	>8 hours	0	0.00%
During a weekday, how many hours do you usually sleep?	4 hours or less	132	18.80%
	5 hours	117	16.70%
	6 hours	117	16.70%
	7 hours	104	14.80%
	8 hours	104	14.80%
	9 hours	65	9.30%
	10 hours or more	63	9.00%
During the last 30 days, how many days have you been bullied?	None	545	77.60%
	1-2 days	92	13.10%
	3-5 days	28	4.00%
	6-9 days	15	2.10%
	10-19 days	11	1.60%
	20-29 days	5	0.70%
	All 30 days	6	0.90%
During the current study year, did you receive classes on how to prevent being bullied?	Yes	249	35.50%
	No	348	49.60%
	I do not know	105	15.00%
During the last 30 days, how many days were you absent due to insecurity or not feeling safe on your way to school?	None	506	72.10%
	One day	37	5.30%
	2-3 days	49	7.00%
	4-5 days	19	2.70%
	6 days or more	91	13.00%

From the analysis, it was observed that three factors had correlation coefficients above 0.2, namely time spent sitting (depression: 0.20, anxiety: 0.13, stress: 0.23), feeling unsafe on the way to school (depression: 0.27, anxiety: 0.29, stress: 0.24), and being bullied in the last 30 days (depression: 0.25, anxiety: 0.31, stress: 0.27). Further analyses revealed significant weak correlations of DASS-21 subscale scores with having at least a fight in the last 12 months, experiencing hunger due to lack of food, experiencing physical assault in the last 12 months, and school year. The detailed results of the correlation coefficients are shown in Table 3.

**Table 3:** Spearman’s correlation of depression, anxiety, and stress with demographic characteristics and social stressors among secondary school students in Saudi Arabia *P-value<0.05; **P-value<0.01

Variable	Depression	Anxiety	Stress
Depression	-	0.701**	0.752**
Anxiety	0.701**	-	0.755**
Stress	0.752**	0.755**	-
Age	-0.005	0.023	0
Body mass index (BMI)	-0.009	-.093*	-0.039
During the last 12 months, how many fights did you have?	0.122**	0.180**	0.144**
During the last 30 days, how many times did you feel hungry because there wasn't enough food at home?	0.111**	0.092*	0.150**
During the usual day, how long do you spend watching TV, using the computer, chatting with friends, or doing something that requires you to be sitting?	0.196**	0.129**	0.231**
During a weekday, how many hours do you usually sleep?	-0.075*	-0.121**	-0.105**
During the last 30 days, how many days have you been bullied?	0.249**	0.306**	0.273**
During the last 12 months, how many times have you been physically assaulted?	0.173**	0.235**	0.226**
During the last 30 days, how many days were you absent due to insecurity or not feeling safe on your way to school?	0.271**	0.285**	0.236**
During a weekday, how many hours do you usually sleep?	-0.075*	-0.121**	-0.105**
During the last 30 days, how many days have you been bullied?	0.249**	0.306**	0.273**

Several significant differences were observed in DASS-21 subscale scores between groups. Females had significantly higher anxiety (P = 0.004) and stress (P = 0.011) scores than males. While not statistically significant, depression was higher among females (P = 0.052). Participants in their first year of school had lower depression (P = 0.015) and stress (P = 0.025) scores than those in their third year, while those in their second year had higher stress (P = 0.025) scores than those in their third year. Students with a normal BMI had lower anxiety (P < 0.001) and stress (P = 0.002) scores than those who were underweight, overweight, or obese. The results have been tabulated in Table 4.

**Table 4:** Comparison of DASS-21 components between the sociodemographic characteristics of secondary school students in Saudi Arabia *Significant association; ^P-value calculated using the Mann-Whitney test; ^^P-value calculated using the Kruskal-Wallis test DASS-21: Depression, Anxiety, and Stress Scale - 21 Items

Variable	Groups	Mean rank		
		Depression	Anxiety	Stress
Gender	Female	359	362.6	361.3
	Male	322.4	308.6	313.5
	P-value^	0.052	0.004*	0.011*
Year	First	303.8	310.1	304.5
	Second	387.5	370.4	383.3
	Third	349.9	353.3	350.8
	P-value^^	0.015*	0.108	0.025*
BMI	Underweight	381.9	404.2	384.2
	Normal	319.5	326.9	324.2
	Overweight	335	301.7	313.4
	Obese	356.6	347.4	375.2
	P-value^^	0.014*	<0.001*	0.002*

Students who were bullied in the last 30 days had significantly higher depression (P < 0.001), anxiety (P < 0.001), and stress (P < 0.001) scores than those who were not bullied. Students who experienced physical assault in the last 12 months had higher depression (P < 0.001), anxiety (P < 0.001), and stress (P < 0.001) scores than those who did not. Participants who had had fights in the last 12 months had higher depression (P = 0.004) and anxiety (P < 0.001) scores than those who did not. Participants who felt unsafe on the way to school had higher depression (P < 0.001), anxiety (P < 0.001), and stress (P < 0.001) scores than those who did not. Finally, participants who reported being hungry due to a lack of food in the last 30 days had higher depression (P = 0.003), anxiety (P = 0.004), and stress (P < 0.001) scores than those who did not report hunger. The related results are shown in Table 5.

**Table 5:** Comparison of DASS-21 components with the presence of social stressors among secondary school students in Saudi Arabia *Significant association; ^P-value calculated using the Mann-Whitney test DASS-21: Depression, Anxiety, and Stress Scale - 21 Items

Statement	Answers	Mean rank		
		Depression	Anxiety	Stress
Being bullied in the last 30 days	Yes	441.8	463.9	452.8
	No	325.5	463.9	322.3
	P-value^	< 0.001*	< 0.001*	< 0.001*
Physical assault in the last 12 months	Yes	454.6	493.2	488.2
	No	340.1	335.8	336.4
	P-value^	< 0.001*	< 0.001*	< 0.001*
Fights in the last 12 months	Yes	389.2	410.2	400
	No	338.5	331.3	334.8
	P-value^	0.004*	< 0.001*	< 0.001*
Absent during the last 30 days due to feeling not safe	Yes	436.3	439.2	424.8
	No	318.6	317.5	323.1
	P-value^	< 0.001*	< 0.001*	< 0.001*
Hungry in the last 30 days because there is no food	Yes	367	366.7	372.6
	No	318.7	319.3	306.7
	P-value^	0.003*	0.004*	< 0.001*

Further analyses included exploring predictors for the total scores of depression, anxiety, and stress. For depression, total scores, male gender, being bullied, physical insult, absence, and hunger were significant predictors with an adjusted prediction coefficient of (R^2^ = 0.12). For anxiety, total scores, male gender, BMI, being bullied, physical insult, and absence were significant predictors with an adjusted prediction coefficient of (R^2^ = 0.17). For stress total scores, male gender, being bullied, physical insult, absence, and hunger were significant predictors with an adjusted prediction coefficient of (R^2^ = 0.13). The results of multiple linear regression are shown in Table 6.

**Table 6:** Multiple linear regression analysis of factors associated with depression, anxiety, and stress total scores among secondary school students *CI: confidence interval; **BMI: body mass index

Predictors	Depression total scores		
	Standardized beta	Unstandardized B (95%CI*)	P-value
Constant		7.1 (5.8, 8.5)	<0.001
Male gender	-0.08	-0.9 (-1.9, -0.04)	0.041
Being bullied in the last 30 days	0.18	2.2 (1.3, 3.2)	<0.001
Physical assault in the last 12 months	0.11	1.9 (0.7, 3.2)	0.003
Absent during the last 30 days due to feeling not safe	0.18	2.1 (1.3, 2.9)	<0.001
Hungry in the last 30 days because there is no food at home	0.06	0.7 (-0.1. 1.5)	0.09
	Anxiety total scores		
	Standardized beta	Unstandardized B (95%CI*)	P-value
Constant		7.8 (6.5, 9.1)	<0.001
Male gender	-0.10	-1.2 (-2, -0.3)	0.008
BMI**	-0.09	-0.5 (-0.8, -0.1)	0.017
Being bullied in the last 30 days	0.21	2.5 (1.6, 3.3)	<0.001
Physical assault in the last 12 months	0.16	2.6 (1.4, 3.8)	<0.001
Absent during the last 30 days due to feeling not safe	0.19	2.1 (1.3, 2.9)	<0.001
	Stress total scores		
	Standardized beta	Unstandardized B (95%CI*)	P-value
Constant		8.3 (6.9, 9.6)	<0.001
Male gender	-0.10	-1.3 (-2.1, -0.4)	0.004
Being bullied in the last 30 days	0.18	2.2 (1.3, 3.1)	<0.001
Physical assault in the last 12 months	0.16	2.7 (1.5, 3.9)	<0.001
Absent during the last 30 days due to feeling not safe	0.13	1.4 (0.6, 2.3)	<0.001
Hungry in the last 30 days because there is no food at home	0.09	0.9 (0.2, 1.8)	0.012

The multiple linear equations for prediction, "$Y= a + \beta X$" can be used for prediction. Where "Y" represents the dependent variable, "a" represents the slope or constant, "β" represents the correlated coefficient of unstandardized B, and X represents the predictor variable. The total scores can be predicted while knowing the values of the predictors. The student's BMI can be multiplied by the coefficient directly, while in the presence of male gender or other binary predictors, the variable coefficient should be multiplied by one. In the case of female gender or absence of the predictor variable, the correlated coefficient should be multiplied by zero. For example, to predict the total scores of anxiety for a female who has a BMI of 24 and has been bullied in the last 30 days, the equation would be "Y= 7.8 + (-1.2×0 + 24×-0.5 + 2.5×1 + 2.6×0 + 2.1×0)". The total score for anxiety as predicted by our model would be (22.3).

The multiple linear equation for prediction, ($Y = a + \beta \times X$\), proves highly useful for predictive purposes. In this context, "Y" represents the dependent variable, "a" represents the slope or constant, "β" denotes the coefficient associated with unstandardized B, and "X" stands for the predictor variable. When it comes to calculating these predictions, the student's BMI can be directly multiplied by the respective coefficient. In instances where binary predictors such as male gender are present, the coefficient of the corresponding variable is simply multiplied by one. Conversely, in cases involving the female gender or the absence of a particular predictor variable, the correlated coefficient is multiplied by zero.

For example, predicting the total anxiety scores for a female student with a BMI of 24 who has experienced bullying within the past 30 days, the equation for this prediction would be: $Y = 7.8 + (-1.2 \times 0) + (24 \times -0.5) + (2.5 \times 1) + (2.6 \times 0) + (2.1 \times 0)$. Consequently, our model predicts a total anxiety score of 22.3 for this case.

## Discussion

This study investigated the prevalence and determinants of depression, anxiety, and stress among secondary school students in Saudi Arabia. The prevalence rates were found to be 35.2% for anxiety, 30.8% for depression, and 14.7% for stress. The results of the present study align with previous studies conducted in the region [1, 14]. However, the present research results also oppose certain prior studies conducted in Saudi Arabia. For instance, a study conducted by Al-Shehri et al. reported a much higher prevalence of depression and anxiety, which was found in 42.9% and 46.6% of secondary school students, respectively. However, the stress percentage found in their study (19%) was quite similar to our findings [18]. On the other hand, lower rates were reported in Jeeluna, a national school-based cross-sectional study that examined the underlying risk factors for feeling very sad or hopeless and for feeling worried among a sample of 12,121 intermediate and secondary school students in Saudi Arabia. The authors utilized a self-administered questionnaire to ascertain the prevalence rates of depression and anxiety, which were found to be 14% and 6%, respectively [19]. Higher rates in our study can be attributed to the difference in the target population, which consisted of only secondary school students.

In the current study, it was found that 7.8% of the students exhibited severe or extremely severe levels of anxiety, whereas 1.3% displayed severe or extremely severe levels of depression. The findings were lower than a previous study conducted in the Qassim region that assessed levels of depression and anxiety in 1,245 students using the Patient Health Questionnaire (PHQ-9) and the General Anxiety Disorder (GAD-7) survey tool. They found that those who were not depressed were 26.0%, the mildly depressed were 34%, and the moderately depressed were 24.6%, whereas 10.4% were moderately severely depressed and 5.0% were severely depressed. In regard to anxiety, 36.5% did not have anxiety, 34.1% had mild anxiety, 19.5% showed moderate anxiety, and 9.8% were classified as having severe anxiety. Similar to our results, a previous study reported that females were higher in number than males in all categories of depression and anxiety [15]. In another study, a total of 545 female students were recruited, and it was found that 73.4% of them exhibited symptoms associated with at least one of the three disorders under investigation. Furthermore, 50.1% of these students presented symptoms indicative of at least two of the studied disorders. The rates of symptom manifestation for depression, anxiety, and stress were found to be 41.5%, 66.2%, and 52.5%, respectively. The majority of symptoms exhibited by individuals were of mild to moderate severity [20].

The present study also revealed a gender disparity in the prevalence of mental health issues, with females reporting higher levels of depression, anxiety, and stress compared to males. Furthermore, in the multiple linear regression, male gender was a predictor for lower scores of depression, anxiety, and stress. These findings resonate with existing literature on gender differences in mental health outcomes during adolescence. A study conducted by Al-Kaabi et al. reported that female students were more likely to experience depression than their male counterparts. Furthermore, they showed that bad personal relationships were the most significant predictors of depression [21]. The greater incidence rates seen in females may be linked to a variety of variables, including heredity, biological factors, psychological factors, and behavioral factors [22]. Similarly, a cross-sectional study conducted on only female participants from Taif reported an estimated prevalence of 42.9%, 54.9%, and 23.1% for depression, anxiety, and obsessive-compulsive symptoms, respectively, and 64.7% had symptoms of the three disorders. Furthermore, the study found that 64.7% of the participants exhibited symptoms associated with all three disorders. The researchers discovered noteworthy positive associations between the score for depression and both the score for anxiety as well as the score for obsessive-compulsive symptoms [23]. Furthermore, comparable to our results, which showed higher scores of depression, anxiety, and stress among females, the Jeeluna study revealed that females exhibited a higher prevalence of symptoms related to depression and anxiety compared to males [20]. In a cross-sectional study comprising 1,723 male students, it was observed that 59.4% of participants exhibited at least one of the three disorders, 40.7% presented with at least two disorders, and 22.6% were diagnosed with all three disorders. Furthermore, it is worth noting that a significant proportion of the participants, specifically 38.2%, exhibited symptoms of depression. In the same study, a considerable percentage of the participants, amounting to 48.9%, reported experiencing anxiety, while 35.5% indicated the presence of stress [1].

In the present study, BMI was significantly related to anxiety; however, BMI did not achieve a statistically significant relationship with depression or stress. In our prediction model, higher BMI was significantly related to lower anxiety scores among secondary school students. While the relationship between higher BMI and the likelihood of depression, anxiety, and stress has been shown in previous studies [24, 25], high BMI might not be the direct cause of depression, anxiety, or stress; rather, it is plausible that other pathways and experiences contribute to the development of depression indirectly. For instance, stressful life events, such as peer victimization and weight-based teasing, could potentially biologically predispose young individuals to depression. Consequently, these factors may serve as contributing factors that lead to depression in obese youth [26].

The current study additionally examined diverse social stressors and their impact on the onset of depression, anxiety, and stress. The findings indicated a statistically significant correlation between experiencing bullying within the past 30 days, being subjected to physical assault within the previous 12 months, and being absent from school due to feelings of insecurity and experiencing symptoms of depression, anxiety, and stress (P<0.001). Among these factors, bullying, physical assault, hunger, and absence due to safety issues were predictors of depression, anxiety, or stress in our linear regression. Previously, a study by AlBuhairan et al. also reported an association between being bullied and depression among Saudi students. Furthermore, they showed that out of 9,073 students, 26% were bullied in the last 30 days, and one out of three experienced physical violence in the past year [27]. Apart from these social stressors, various other factors, such as poor relationships with peers and family, not being happy with body image, emotional abuse, and physical abuse, have been implicated in the development of anxiety and depression [14].

One of the strengths of this study is its large sample size and the use of standardized assessment tools, namely, DASS-21 to measure depression, anxiety, and stress, and the GSHS self-administered questionnaire developed by the WHO to measure health behavior. The study also considered several sociodemographic and social stressor variables, providing a comprehensive analysis of the factors associated with mental health outcomes in this population. However, there are some limitations to this study. For instance, data on potential confounding variables, such as family history of mental illness or prior mental health treatment, were not included. Moreover, the predominant representation of females in the sample could have potentially resulted in over-reporting of symptoms and consequently inflated the prevalence rates of depression, anxiety, and stress.

## Conclusions

This study aimed to investigate the prevalence and determinants of depression, anxiety, and stress among secondary school students in Saudi Arabia. The results indicate that anxiety had the highest prevalence at 35.2%, followed by depression at 30.8% and stress at 14.7%. Additionally, the study found that females had higher depression, anxiety, and stress scores than males. Furthermore, students who experienced bullying, physical assault, and fights had higher depression, anxiety, and stress scores than those who did not. Time spent sitting was also found to be moderately correlated with depression, anxiety, and stress.

The findings suggest that schools and policymakers should prioritize creating safe and supportive environments for students, providing resources and support for students experiencing mental health issues, and implementing preventive interventions to reduce the prevalence of these disorders. Future research should investigate the effectiveness of interventions aimed at reducing the prevalence of mental health disorders among secondary school students in Saudi Arabia.

## Appendices

Appendix A

The Depression, Anxiety, and Stress (DASS-21) Tool

**Table 7:** Depression, Anxiety, and Stress Scale - 21 Items (DASS-21) [17]

N	Item	Did not apply to me at all	Applied to me to some degree, or some of the time	Applied to me to a considerable degree or a good part of the time	Applied to me very much or most of the time
1 (s)	I found it hard to wind down	0	1	2	3
2 (a)	I was aware of the dryness of my mouth	0	1	2	3
3 (d)	I couldn’t seem to experience any positive feelings at all	0	1	2	3
4 (a)	I experienced breathing difficulty (e.g. excessively rapid breathing, breathlessness in the absence of physical exertion)	0	1	2	3
5 (d)	I found it difficult to work up the initiative to do things	0	1	2	3
6 (s)	I tended to over-react to situations	0	1	2	3
7 (a)	I experienced trembling (e.g. in my hands)	0	1	2	3
8 (s)	I felt that I was using a lot of nervous energy	0	1	2	3
9 (a)	I was worried about situations in which I might panic and make a fool of myself	0	1	2	3
10 (d)	I felt that I had nothing to look forward to	0	1	2	3
11 (s)	I found myself getting agitated	0	1	2	3
12 (s)	I found it difficult to relax	0	1	2	3
13 (d)	I felt down-hearted and blue	0	1	2	3
14 (s)	I was intolerant of anything that kept me from getting on with what I was doing	0	1	2	3
15 (a)	I felt I was close to panic	0	1	2	3
16 (d)	I was unable to become enthusiastic about anything	0	1	2	3
17 (d)	I felt I wasn’t worth much as a person	0	1	2	3
18 (s)	I felt that I was rather touchy	0	1	2	3
19 (a)	I was aware of the action of my heart in the absence of physical exertion (e.g. sense of heart rate increase, heart missing a beat)	0	1	2	3
20 (a)	I felt scared without any good reason	0	1	2	3
21 (d)	I felt that life was meaningless	0	1	2	3

Appendix B

The DASS-21 Scoring Instructions

Each of the three DASS-21 scales contains seven items, divided into subscales with similar content. Recommended cut-off scores for conventional severity are as according to Table 8. Scores on the DASS-21 will need to be multiplied by two to calculate the final score.

**Table 8:** Recommended cut-off scores for conventional severity labels (normal, mild, moderate, severe, extremely severe)

	Depression	Anxiety	Stress
Normal	0-9	0-7	0-14
Mild	10-13	8-9	15-18
Moderate	14-20	10-14	19-25
Severe	21-27	15-19	26-33
Extremely Severe	28+	20+	34+
